# Predicted cardiovascular disease risk and prescribing of antihypertensive therapy among patients with hypertension in Australia using MedicineInsight

**DOI:** 10.1038/s41371-022-00691-z

**Published:** 2022-05-02

**Authors:** Jacqueline Roseleur, David A. Gonzalez-Chica, Jonathan Karnon, Nigel P. Stocks

**Affiliations:** 1 School of Public Health, Faculty of Health Sciences, The University of Adelaide, Adelaide, SA Australia; 2 Discipline of General Practice, Adelaide Medical School, The University of Adelaide, Adelaide, SA Australia; 3 Flinders Health and Medical Research Institute, College of Medicine and Public Health, Flinders University, Bedford Park, Adelaide, SA Australia; 4 Adelaide Rural Clinical School, The University of Adelaide, Adelaide, SA Australia

**Keywords:** Hypertension, Risk factors, Preventive medicine

## Abstract

Hypertension guidelines recommend that absolute cardiovascular disease (CVD) risk guide the management of hypertensive patients. This study aimed to assess the proportion of patients with diagnosed hypertension with sufficient data to calculate absolute CVD risk and determine whether CVD risk is associated with prescribing of antihypertensive therapies. This was a cross-sectional study using a large national database of electronic medical records of patients attending general practice in 2018 (MedicineInsight). Of 571,492 patients aged 45–74 years without a history of CVD, 251,733 [40.6% (95% CI: 39.8–41.2)] had a recorded hypertension diagnosis. The proportion of patients with sufficient recorded data available to calculate CVD risk was higher for patients diagnosed with hypertension [51.0% (95% CI: 48.0–53.9)] than for patients without a diagnosis of hypertension [38.7% (95% CI: 36.5–41.0)]. Of those patients with sufficient data to calculate CVD risk, 29.3% (95% CI: 28.1–30.6) were at high risk clinically, 6.0% (95% CI: 5.8–6.3) were at high risk based on their CVD risk score, 12.8% (95% CI: 12.5–13.2) at moderate risk and 51.8% (95% CI: 50.8–52.9) at low risk. The overall prevalence of antihypertensive therapy was 60.9% (95% CI: 59.3–62.5). Prescribing was slightly lower in patients at high risk based on their CVD risk score [57.4% (95% CI: 55.4–59.4)] compared with those at low [63.3% (95% CI: 61.9–64.8)] or moderate risk [61.8% (95% CI: 60.2–63.4)] or at high risk clinically [64.1% (95% CI: 61.9–66.3)]. Guideline adherence is suboptimal, and many patients miss out on treatments that may prevent future CVD events.

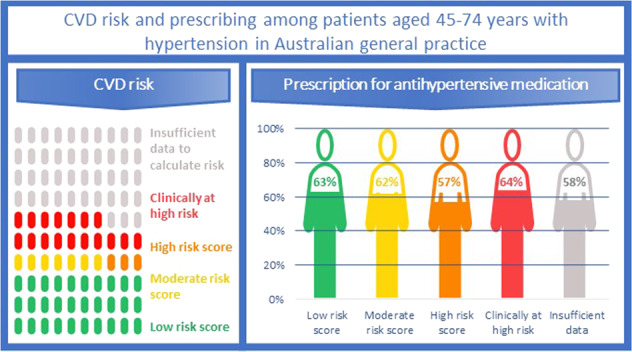

## Introduction

Traditionally, hypertension guidelines have relied exclusively on blood pressure (BP) levels to guide treatment initiation and intensity [1]. However, cardiovascular disease (CVD) risk factors tend to cluster together, particularly in patients with hypertension. Moreover, antihypertensive treatment according to CVD risk is more effective [2] and cost-effective [3] than using BP levels alone. Therefore, guidelines now recommend that management and prevention of hypertension should also consider absolute CVD risk [4–6].

In Australia, the Heart Foundation recommends calculating the absolute risk of a primary CVD event over the next 5 years by applying the Australian National Vascular Disease Prevention Alliance (NVDPA) risk assessment and risk management algorithm [4], which includes the Framingham CVD risk equation [7]. The risk assessment applies to adults aged 45 and 74 years without a known history of CVD. In Aboriginal and Torres Strait Islander peoples, adults aged between 35 and 74 years are eligible [4, 8]. Patients at low or moderate CVD risk should be prescribed lifestyle therapy and, depending on BP levels, treated with an antihypertensive agent. Patients at high absolute CVD risk should always be managed with antihypertensives [4].

Existing evidence suggests that between 41 and 96% of physicians use a CVD risk calculator to assess absolute CVD risk [9–12]. These studies investigated absolute CVD risk assessment in all eligible patients, not only those with hypertension. As hypertension is already considered a risk factor for CVD [4], it is expected that a high proportion of patients with hypertension would have their absolute CVD risk assessed.

Studies evaluating the CVD risk profile of patients with hypertension in primary care are limited [13–15]. A study in Spain aimed to define the CVD risk profile of patients diagnosed with hypertension in primary and specialist care [15]. Even though physicians were required to undertake a complete medical history and a physical examination as part of the data collection process, 22% of patients had insufficient data to calculate CVD risk. Similarly, a more recent Swiss study requiring general practitioners (GPs) to record demographic and clinical data and conduct laboratory screening for lipid and plasma glucose levels, found that 13% of patients had missing data for determining dyslipidaemia status [13]. In Korea, Kim et al. assessed the CVD risk profile using measures collected as part of the study and the prescribing of antihypertensive therapy in patients with hypertension [14]. The authors reported no missing data and found that treatment with antihypertensive therapy did not differ by CVD risk status.

Evidence from real-world primary care settings where GPs are not prompted to collect data as part of a study is lacking. However, electronic health records (EHRs) provide an opportunity to evaluate real-world practice of CVD risk assessment in patients diagnosed with hypertension and subsequent prescribing patterns [16]. Therefore, this study aimed to (1) assess whether a higher proportion of patients with diagnosed hypertension aged 45–74 years and regularly attending Australian general practice have sufficient data in their EHR to calculate absolute CVD risk compared with patients without a diagnosis of hypertension, (2) determine whether a higher CVD risk is associated with more frequent prescribing of antihypertensive therapies among patients with hypertension aged 45–74, and (3) assess whether these findings vary according to age and sex.

## Methods

This study is a cross-sectional analysis of MedicineInsight, a large Australian general practice database of EHR. This study was reported according to REporting of studies Conducted using Observational Routinely-collected health Data Statement reporting guidelines [17].

### Data source

As at October 2018, MedicineInsight included data from patients attending over 2700 GPs and 660 general practices across all states and territories (8.2% of all Australian practices) [18] and has been widely used to investigate diverse acute and chronic conditions [19–21]. Patients in the database are comparable, but not representative, to the general population as measured by sociodemographic variables and clinical conditions [18]. Details of the data collection process are published elsewhere [18]. In summary, de-identified EHRs from patients are collected monthly and include diagnoses, reasons for encounters, prescriptions, immunisations, clinical measurements (e.g. BP, pulse, weight), laboratory test orders and results and patient sociodemographic information. Patients within each practice receive a unique identification number that allows the patient to be followed over time. Extraction algorithms for identifying chronic condition diagnoses have recently been validated [22].

### Study population

This study includes all patients aged between 45 and 74 years, or between 35 and 74 years for Aboriginal and Torres Strait Islander people, without CVD recorded in their EHR (i.e. ischaemic heart disease, heart failure, stroke, peripheral artery disease and aortic disease) who regularly attended one of these practices (i.e. at least three visits between 2016 and 2018) [23]. The methods used to identify patients diagnosed with hypertension have been described elsewhere [24]. Briefly, GPs can record clinical data (diagnosis, reason for encounter, reason for prescription) with either pre-coded terms or free-text. Misspellings, abbreviations, synonyms or spelling variations are common, and we consequently used a range of terms for “hypertension” to account for these variations. All available data in the patient’s EHR was reviewed to identify those with hypertension diagnosis. Almost 70% of patients had data available since 2011. Patients were considered to have hypertension if (1) the condition was recorded as a diagnosis, reason for encounter or reason for prescription (Supplementary File), or (2) if the patient received a prescription for antihypertensive therapy preceded by an elevated BP (i.e. BP higher than 140/90 mmHg). By including an elevated BP, we aimed to reduce misclassification of patients taking antihypertensive therapy for conditions other than hypertension (e.g. heart failure, myocardial infarction). Antihypertensive medications included angiotensin-converting enzyme inhibitors and angiotensin II receptor blockers (Anatomical Therapeutic Chemical (ATC) C09), beta-blockers (ATC C07), calcium channel blockers (ATC C08), diuretics (ATC C03) and alpha-blockers (ATC C02).

### Data and variables

#### Cardiovascular risk

First, we estimated whether patients had available recorded information on the different risk factors to enable CVD risk calculation, irrespective of whether they had hypertension or not. We used the most recent measures recorded (i.e. systolic BP, total cholesterol, HDL cholesterol, albumin:creatinine ratio and estimated glomerular filtration rate). As guidelines recommend CVD risk be reviewed at least every 2 years [8], measures recorded in 2015 were also considered to accommodate patients who last visited their GP in 2017. Hence, measures between 2015 and 2018 were included. Where smoking status was not recorded, patients were assumed to be non-smokers. A comparison of the proportion of patients recorded as smokers with data reported in the National Drug Strategy Household Survey found similar proportions for all age groups by sex [25]. As left ventricular hypertrophy is challenging to identify in the EHR, we assumed left ventricular hypertrophy was absent for all patients.

Thereafter, for patients with a diagnosis of hypertension, we followed the recommendations of the National Heart Foundation of Australia guidelines [4] and classified patients with the following conditions as clinically at high risk of CVD: (1) people with diabetes and over 60 years of age, (2) those with diabetes and microalbuminuria (albumin:creatinine ratio >3.5 for females and >2.5 for males), (3) patients with moderate or severe chronic kidney disease [estimated glomerular filtration rate below 45 ml/min/1.73 m^2^ or persistent proteinuria (albumin:creatinine ratio >35 mg/mmol in females and >25 mg/mmol in males—two positive measurements, 3 months apart)], (4) systolic BP above 180 mmHg, (5) diastolic BP above 110 mmHg, (6) familial hypercholesterolaemia or (7) total cholesterol level exceeding 7.5 mmol/l [4, 8]. Patients were considered to have diabetes when the patient record had either a diagnosis, encounter reason or prescription reason of diabetes, or they were prescribed antidiabetic medication (ATC A10; except for those with a diagnosis of polycystic ovarian syndrome). Similarly, patients were considered to have familial hypercholesterolaemia when the patient record had either a diagnosis, encounter reason or prescription reason of familial hypercholesterolaemia.

Next, for those patients with diagnosed hypertension who were not at high risk clinically and with sufficient variables available, we calculated the absolute risk of a primary CVD event over the next 5 years by applying the Australian NVDPA risk assessment and risk management algorithm [4, 8]. The absolute CVD risk was categorised as low (<10%), moderate (10–15%), or high (>15%). The NVDPA algorithm underestimates risk in Aboriginal and Torres Strait Islander patients and recommends adding 5% to the calculated risk score [4, 26]. By using that approach, 706 out of 1578 and 317 out of 317 Aboriginal and Torres Strait Islander patients were reclassified from low to moderate risk and from moderate to high risk, respectively.

Therefore, except for those with insufficient data for CVD risk score calculation, patients were classified in one of four groups: (1) low CVD risk, (2) moderate CVD risk, (3) high CVD risk (based on the NVDPA algorithm), or (4) clinically at high risk of CVD.

#### Outcome: guideline-recommended therapy

According to current guidelines, patients at low or moderate CVD risk should be prescribed lifestyle therapy and, depending on BP levels (e.g. BP persistently ≥160/90 mmHg), an antihypertensive agent. In contrast, patients at high CVD risk should be treated with an antihypertensive agent irrespective of their BP levels [4]. To assess compliance with these recommendations, the history of antihypertensive prescription in the last 6 months (July to December 2018) was examined. For comparison, we also examined the prescription history of patients with insufficient data available for CVD risk calculation. We also considered alternative prescribing investigation periods [12 months (January to December 2018) and 24 months (January 2017 to December 2018)] to explore changes in prescribing patterns over time.

#### Covariates

Patient sociodemographic characteristics included sex (male/female), 5-year age groups, remoteness (major cities, inner regional and outer regional/remote and very remote) and socioeconomic status in quintiles, as measured by the Index of Relative Socioeconomic Advantage and Disadvantage (IRSAD). Age in 2018 was calculated using the patient’s year of birth. IRSAD is a macroeconomic indicator of relative advantage and disadvantage developed by the Australian Bureau of Statistics that summarises information about households’ social and economic conditions within an area (i.e. income, education, employment, occupation and housing characteristics) and is based on residential postcodes [27].

### Statistical methods

All analyses were performed in STATA 16.0 (StataCorp, College Station, Texas, USA), using practices as clusters and conditioned on the number of consultations to minimise selection bias (i.e. the likelihood of receiving medical treatments or diagnosis increase with the number of visits to the practice) [28].

The sociodemographic and cardiovascular risk characteristics were presented by sex and age group and expressed as proportions (%), with their corresponding 95% confidence intervals (95% CIs). We present crude results unless otherwise stated.

The proportions of patients prescribed antihypertensives according to CVD risk status (low, moderate, high, or high risk clinically) or those with insufficient data to calculate CVD risk was assessed using logistic regression adjusted for age, IRSAD and remoteness, and was presented separately for males and females.

The Human Research Ethics Committee of the University of Adelaide exempted this study for ethical review, as it used existing and non-identifiable data. Access to the data for this study was approved by the MedicineInsight Data Governance Committee (project 2016-007).

## Results

### Hypertension diagnosis and data availability

**Fig. 1:**
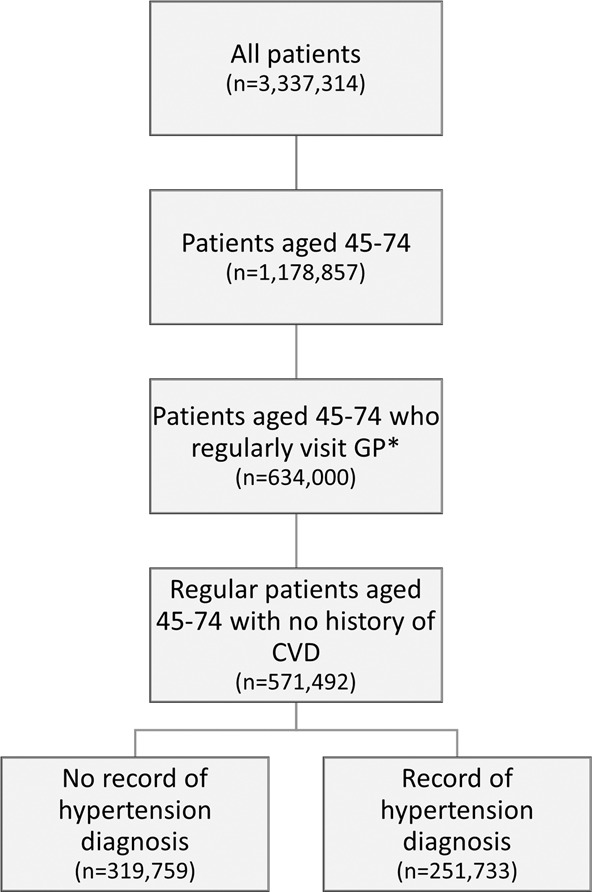
Flow diagram describing the derivation of the study cohort. *Regular attendance defined as at least three visits in two consecutive years between 2016 and 2018.

The sample included 571,492 regular patients without a history of CVD and aged 45–74 years [mean age 58.8 years (SD 8.6), 57.7% female], including 12,129 Aboriginal and Torres Strait Islander people aged 35–74 years. Of these, 251,733 [40.6% (95% CI: 39.8–41.2)] had a recorded diagnosis of hypertension [mean age 62.3 years (SD 8.0), 47.9% female]. Figure 1 describes the derivation of the study cohort.

**Fig. 2:**
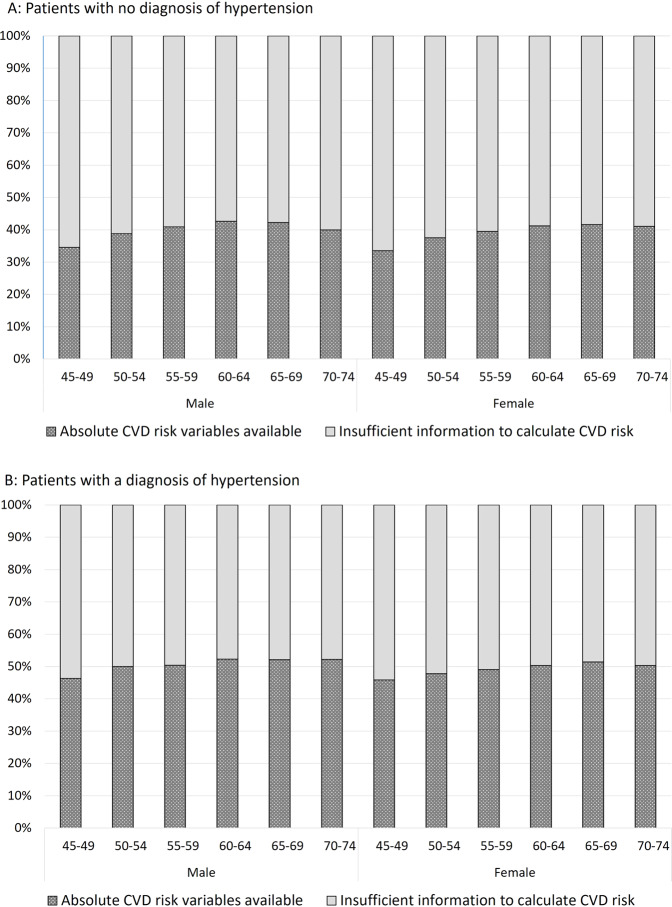
Data availability for CVD risk calculation. Distribution of the availability of data to calculate absolute risk of a primary CVD event over the next 5 years for patients without a history of cardiovascular disease aged between 45 and 74 by age group and sex for patients (**A**) with no diagnosis of hypertension (*n* = 319,719) and (**B**) patients with a diagnosis of hypertension (*n* = 251,733).

The proportion of patients with sufficient recorded data available to calculate CVD risk was higher for patients diagnosed with hypertension [51.0% (95% CI: 48.0–53.9)] than for patients without a diagnosis of hypertension [38.7% (95% CI: 36.5–41.0)]. This finding was consistent for males and females across all age groups (Fig. 2).

### Characteristics of patients diagnosed with hypertension

In those with a hypertension diagnosis, there were no differences according to age, sex, or Indigenous status between those with sufficient (*n* = 128,836) or insufficient (*n* = 122,897) data to calculate their CVD risk (Table 1). However, a higher proportion of patients with sufficient data were from regional/remote/very remote areas (18.2% vs. 10.7%), the lowest IRSAD quintile (21.9% vs. 16.4%), had a diagnosis of diabetes (23.7% vs. 18.9%) or chronic kidney disease (3.0% vs. 2.4%) than those with insufficient data.

**Table 1:** Characteristics of patients aged 45–74 years with a diagnosis of hypertension and no history of CVD with sufficient and insufficient data to calculate absolute CVD risk (*n* = 251,733).

	Sufficient data to calculate CVD risk (*n* = 128,836)		Insufficient data to calculate CVD risk (*n* = 122,897)	
Characteristic	%	95% CI	%	95% CI
Age (Mean, SD)	62.0 (8.0)		61.6 (8.2)	
Sex				
Female	49.9	[49.3–50.5]	51.2	[50.5–52.0]
IRSAD quintile				
Highest	20.3	[17.1–24.0]	22.4	[18.7–26.5]
2nd upper	16.8	[14.7–19.1]	17.4	[15.0–20.0]
Intermediate	23.1	[20.0–26.5]	24.3	[20.6–28.4]
2nd lower	17.0	[14.4–20.1]	18.9	[15.8–22.3]
Lowest	21.9	[18.1–26.3]	16.4	[13.2–20.3]
Not recorded	0.9	[0.6–1.2]	0.7	[0.5–0.9]
Remoteness				
Major cities	53.3	[47.7–58.8]	60.0	[54.3–65.5]
Inner regional	27.9	[23.4–32.9]	28.8	[24.0–34.1]
Outer regional/remote/very remote	18.2	[14.2–23.2]	10.7	[8.2–13.9]
Not recorded	0.6	[0.4–0.8]	0.4	[0.3–0.5]
Indigenous status				
Neither Aboriginal nor Torres Strait Islander	80.7	[77.6–83.4]	81.9	[79.2–84.4]
Aboriginal and/or Torres Strait Islander	2.2	[1.8–2.7]	2.0	[1.7–2.2]
Not stated	17.2	[14.4–20.3]	16.1	[13.6–18.9]
Smoking status				
Non smoker	51.4	[50.5–52.3]	50.7	[49.8–51.7]
Smoker	11.8	[11.3–12.4]	12.5	[12.0–13.1]
Ex smoker	32.8	[32.1–33.5]	30.5	[29.7–31.3]
Not recorded	4.0	[3.5–4.5]	6.2	[5.5–7.1]
Blood pressure grade^a^				
Controlled	52.8	[52.0–53.6]	49.4	[48.6–50.1]
Grade 1	36.5	[36.0–37.1]	36.8	[36.2–37.3]
Grade 2	9.0	[8.7–9.3]	9.6	[9.3–9.9]
Grade 3	1.6	[1.5–1.7]	1.8	[1.7–2.0]
Not recorded	0.0	[0.0–0.0]	2.5	[2.0–3.0]
Diabetes	23.7	[22.9–24.5]	18.9	[18.4–19.5]
Chronic kidney disease^a,b^	3.0	[2.7–3.3]	2.4	[2.2–2.7]
Familial hypercholesterolaemia	0.2	[0.2–0.2]	0.1	[0.1–0.1]

Crude results presented. Percentages and 95% CI were estimated considering the clusters (general practices) and the individual’s probability of being in the sample.

^a^Only measures recorded between 2015 and 2018 were used.

^b^Patients with record of a diagnosis of chronic kidney disease or an estimated glomerular filtration rate <45 ml/min/1.73 m^2^ or persistent proteinuria.

Supplementary Table 1 shows the socioeconomic characteristics and cardiovascular risk factors by age group and sex for those with diagnosed hypertension. More males were current smokers (13.4% vs. 11.0%, *p* < 0.001) or had diabetes (23.1% vs. 19.7%, *p* < 0.001) than females. The mean systolic and diastolic BP for males and females were similar [137.9 (SD 15.3)/81.5 (SD 10.3) mmHg and 136.2 (SD 16.4)/81.0 (SD 10.5) mmHg, respectively]. The mean total cholesterol for males and females was 4.9 (SD 1.1) mmol/l and 5.3 (SD 1.1) mmol/l, respectively and the mean HDL cholesterol was 1.3 (SD 0.4) mmol/l for males and 1.6 (SD 0.4) mmol/l for females.

### Hypertension and cardiovascular disease risk

**Fig. 3:**
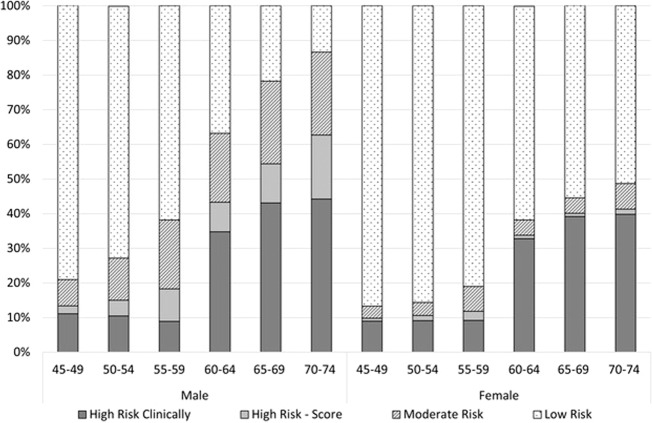
Distribution of absolute risk of a primary CVD event over the next 5 years for patients with a diagnosis of hypertension aged 45–74, no history of CVD and sufficient details to calculate risk if not at high risk clinically, by age group and sex (*n* = 146,655).

Apart from those patients with hypertension and sufficient data to calculate their CVD risk (*n* = 128,836), another 17,819 patients with hypertension were identified as clinically at high risk of CVD [4] despite having insufficient data for risk estimation. These patients were included in the sample for further analyses. Therefore, of all these patients with hypertension (*n* = 146,655), 29.3% (95% CI: 28.1–30.6) were at high risk clinically, 6.0% (95% CI: 5.8–6.3) were at high risk based on their CVD risk score, 12.8% (95% CI: 12.5–13.2) at moderate risk and 51.8% (95% CI: 50.8–52.9) at low risk. Figure 3 shows that similar proportions of males [30.1% (95% CI: 28.8–31.5)] and females [28.5% (95% CI: 27.2–29.7)] were at high risk clinically. However, based on the CVD risk calculation, a larger proportion of males than females were at high risk (10.6% vs. 1.4%, *p* < 0.001) or moderate risk (20.1% vs. 5.5%, *p* < 0.001). This difference was larger in older age groups (Fig. 3).

### Prescribing of guideline-recommended therapy

**Fig. 4:**
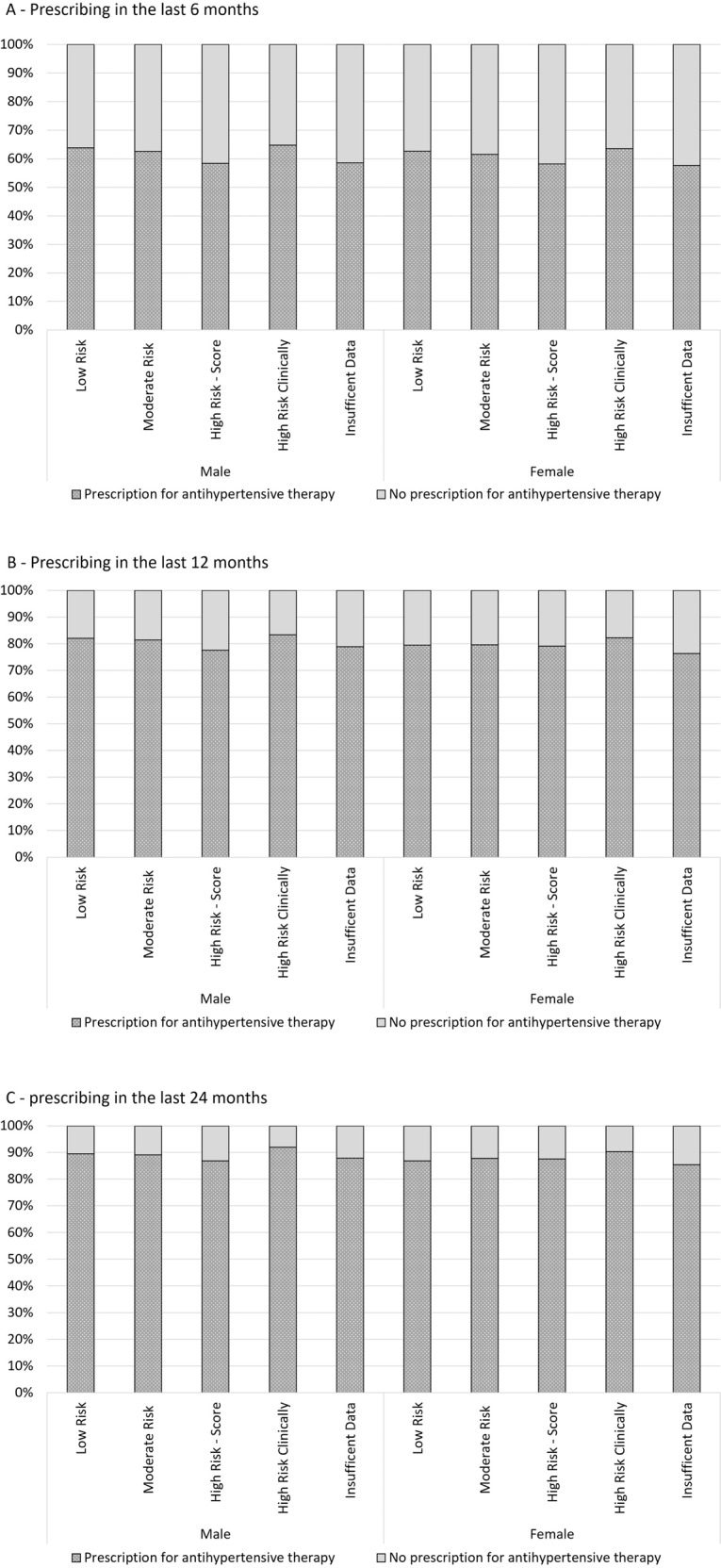
Antihypertensive therapy prescribing by CVD risk. Prescribing of antihypertensive therapy by absolute risk of a primary CVD event over the next 5 years and those with insufficient data to calculate CVD risk, and sex, in the past 6 months (**A**), 12 months (**B**) and 24 months (**C**) for patients aged 45–74 years (*n* = 251,733).

The overall prevalence of antihypertensive therapy in males and females was 61.3% (95% CI: 59.7–62.9) and 60.5% (95% CI: 58.9–62.1), respectively. Figure 4A presents the proportion of patients prescribed an antihypertensive therapy in the last 6 months by CVD risk and sex. Overall, prescribing was slightly lower in patients at high risk based on their CVD risk score [57.4% (95% CI: 55.4–59.4)] compared with those at low [63.3% (95% CI: 61.9–64.8)] or moderate risk [61.8% (95% CI: 60.2–63.4)] or at high risk clinically [64.1% (95% CI: 61.9–66.3)], and this pattern was similar for males and females. For comparison, Fig. 4A also shows prescribing of antihypertensive therapy for patients with hypertension but with insufficient data to calculate CVD risk. Prescribing for these patients was similar to those at high risk based on their CVD risk score, with 58.5% (95% CI: 56.6–60.5) for males and 57.6% (95% CI: 55.6–59.6) for females.

When the prescribing investigation periods were extended to 12 and 24 months (Fig. 4B, C), higher proportions of patients in all risk categories were prescribed antihypertensive therapy. However, the prevalence of antihypertensive prescriptions was similar across all CVD risk categories.

## Discussion

Two main findings can be highlighted in this study. First, CVD risk calculation was only possible in half of the patients with hypertension, and neither age, sex, smoking status, nor BP grade influenced data availability. Second, our findings indicate that GP prescribing of antihypertensive therapy was not solely guided by absolute CVD risk.

### CVD risk assessment

Despite the recommendation by Heart Foundation guidelines that management of hypertension be based on absolute CVD risk, two Australian studies have found that GPs still base their treatment and management of hypertension on BP as a single risk factor [29, 30]. Jansen et al. [30] conducted an experimental study in 2012 with 144 GPs to understand the use of individual risk factors and absolute CVD risk in making decisions about patient management. GPs stated that they would prescribe BP medication for 93% of the cases with high absolute CVD risk and 83% of the cases with lower absolute risk. More recently, Chapman et al. [29] conducted interviews and focus group discussions with 18 GPs on determining the role of BP in the management of absolute CVD risk. They found that when GPs were provided with absolute CVD risk data, all GPs tended to use single risk factor management strategies. Our study, which explored the EHRs of more than 570,000 regular patients, supports these findings. We found that CVD risk calculation was only possible in half of the patients with hypertension, indicating that absolute CVD risk was unavailable to guide the management of patients. This proportion was higher than those without a hypertension diagnosis (51.0% vs. 38.7%), but still suboptimal.

Surveys of GPs report high levels of awareness of CVD risk calculators (92–96%) [12, 31], but considerable variation in the use of CVD risk calculators (41–96%) [9–12, 31]. Among those who reported using these calculators, use was inconsistent. In the US, only 19% of physicians reported always or nearly always using CVD risk when considering primary prevention [12]. In Australia, half of GPs reported assessing CVD risk in more than 80% of their patients [31]. These findings are supported by studies analysing EHRs that found varying rates of missing data to calculate CVD risk. For example, estimates for missing cholesterol data ranged from 31 to 78% [32–34], which is consistent with the findings of our study that almost 50% of patients did not have a cholesterol measure recorded in their EHR.

In Australia, patients aged 45–49 years with at least one identifiable risk factor (lifestyle, biomedical or family history) are eligible for a Medicare-funded health check. This programme aims to prevent or delay the onset of chronic disease and includes undertaking examinations and investigations as clinically required [8]. Furthermore, the Royal Australian College of General Practitioners Guidelines for Preventative Activities in General Practice recommends assessing lipid levels from 45 years [35]. In our study, only 46% of patients aged 45–49 with diagnosed hypertension had sufficient data to calculate CVD risk, despite hypertension being a biomedical risk factor for chronic disease and the existence of a government-funded programme that promotes preventive activities in that age group. This finding suggests that compliance with preventive recommendations is suboptimal, even when a funding mechanism is available. To address the underuse of CVD risk assessment, the Australian Government introduced additional funding in 2019 to fund CVD risk assessment and ongoing management for all patients aged 30 years and older [36]. Further studies are necessary to investigate the impact of this measure.

### Prescribing of guideline-recommended therapy

Our study found that GP prescribing of antihypertensive therapy was not guided solely by absolute CVD risk. Approximately the same proportion (~61%) of patients diagnosed with hypertension in all risk groups, including patients with insufficient data available for CVD risk assessment, had been prescribed an antihypertensive drug in the last 6 months of the study. This pattern was similar for males and females. We are only aware of one other study investigating the prescribing patterns by CVD risk in a population with diagnosed hypertension [14]. This Korean study also found no difference in recorded prescribing patterns across risk groups. Australian studies have investigated prescribing by CVD risk in the general population, rather than in those with diagnosed hypertension [37, 38]. Using data from a national survey, Banks et al. found that individuals at high CVD risk were more likely to be taking antihypertensive therapy than those at low CVD risk [37]. As our study included only patients diagnosed with hypertension who regularly visited their GP, we cannot compare our findings to national survey data, which included individuals who may be different to those who regularly consult with their GP. The Australian Hypertension and Absolute Risk Study (AusHEART) study described the CVD risk and prescribing patterns of patients aged 55 years or older attending general practice [38]. This study calculated the absolute CVD risk of patients using the Framingham risk equations and three different guideline adjustments, including the NVDPA. Prescribing of antihypertensive therapy by CVD risk increased with higher risk levels. However, prescribing data were only presented for risk categories calculated using the National Heart Foundation 2004 guidelines. These guidelines categorise a higher proportion of patients as high risk than the NVDPA, once again limiting our ability to make comparisons.

The prescribing of antihypertensive medications in our study did not vary by absolute CVD risk, even when we investigated longer prescribing periods. These results reflect undertreatment of high-risk patients and potential overtreatment of low-risk patients for CVD risk reduction. According to the Australian guidelines [4], all patients at high risk should be prescribed antihypertensive therapy to reduce their CVD risk. In our study, ~77% of patients with hypertension at high CVD risk and without a prescription of antihypertensive therapy were above target BP levels [i.e. 140/90 mmHg; mean BP in this group 148.0/83.9 mmHg (SD 13.6/9.7); data not shown in tables]. On the other hand, 41% of those at low CVD risk and not managed with antihypertensives had BP levels above the threshold. Clinical inertia (i.e. failure to initiate or escalate treatment when indicated) is often cited as a reason for patients not receiving prescriptions for guideline-recommended therapy [39]. However, debates around inappropriate therapeutic inertia and appropriate inaction continue [40]. Lebeau et al. recently undertook a consensus study to create operational definitions for appropriate inaction and inappropriate inertia in managing patients with hypertension in primary care [40]. Appropriate inaction was defined as not initiating or intensifying treatment for a patient for whom BP goals defined by guidelines have not been achieved and when at least one of the following conditions occurs: (1) elevated BP has not been confirmed by self-measurement or ambulatory BP monitoring, (2) there is a legitimate reservation regarding the reliability of the measurements, (3) there is an adherence concern regarding pharmacological therapy, (4) there is a specific iatrogenic risk, specifically for orthostatic hypotension in the elderly, (5) there is another medical priority more critical and more urgent, and (6) access to treatment is challenging. This definition addresses some of the limitations of previous clinical inertia definitions by recognising the complexity of the GP-patient relationship. Whether appropriate inaction explains our findings is uncertain and would require further research.

### Strengths and limitations

Our study is the first in Australia to evaluate the cardiovascular risk profile of patients diagnosed with hypertension and included a large sample of patients across Australian general practice. A limitation of MedicineInsight is the inability to track patients across different practices. This limitation may have resulted in underestimating the proportion of patients with available data to calculate CVD risk. However, the median number of annual visits between 2016 and 2018 ranged from six for those with insufficient data to eight for those clinically at high risk. Therefore, most patients visited the same practice every 2 months on average, enabling enough opportunities to have their CVD risk assessed. Another limitation of MedicineInsight is that progress notes are not extracted, and these may contain information related to CVD risk factors. However, it is unlikely that this limitation would change our results. A recent validation study found accuracy close to 90% between algorithms for chronic conditions using the same fields as our study compared to the original EHR, which included the progress notes [22]. The Framingham CVD risk equation is intended for treatment-naive individuals and will underestimate risk in those receiving treatment. As we used the most recent BP and cholesterol measures to calculate CVD risk, those classified as low or moderate absolute CVD risk may have had a higher baseline risk before medication initiation.

## Conclusions

Patients with hypertension are at increased risk of CVD. Despite guidelines recommending the use of absolute CVD risk in the management of hypertension, only half of patients have sufficient data to calculate CVD risk. For hypertensive patients aged 45–74 who regularly visit their GP, with a calculated high or moderate CVD risk, 40% were not prescribed antihypertensive therapies, despite three-quarters of them having BP levels above the threshold. Therefore, many patients miss out on guideline-promoted treatments that minimise BP complications and reduce the risk of future CVD events.

## Summary table

### What is known on this topic

- Cardiovascular disease (CVD) risk factors tend to cluster together, particularly in patients with hypertension.
- Guidelines therefore recommend that the management of hypertension should be guided by absolute CVD risk, rather than blood pressure alone.
- Electronic health records provide an opportunity to evaluate real-world practice of CVD risk assessment and subsequent prescribing patterns.

### What this study adds

- CVD risk calculation was only possible in half of patients with a diagnosis of hypertension.
- GP prescribing of antihypertensive therapy was not solely guided by absolute CVD risk.
- Many patients miss out on guideline-promoted treatments that minimise BP complications and reduce the risk of future CVD events.

## Supplementary information

Supplementary File

Supplementary Table

## Data Availability

Data may be obtained from MedicineInsight and are not publicly available. Third parties may express an interest in the information collected through MedicineInsight. The provision of information in these instances undergoes a formal approval process and is guided by the MedicineInsight independent external Data Governance Committee. This Committee includes general practitioners, consumer advocates, privacy experts and researchers.
