# Supplementary material for: Predicted cardiovascular disease risk and prescribing of antihypertensive therapy among patients with hypertension in Australia using MedicineInsight
Source: J Hum Hypertens. 2022 May 2;37(5):370–8. doi: 10.1038/s41371-022-00691-z (PMC10156591; doi:10.1038/s41371-022-00691-z)
Supplement: Supplementary file 1 — Supplementary File [file 41371_2022_691_MOESM1_ESM.docx]

**Supplementary File**

**Terms used to identify a diagnosis of hypertension in the electronic health record**

Coded as diagnosis of hypertension if the diagnosis, reason for encounter or reason for prescription fields contained the following terms:

HYPERTE* | HYPERTANSION | HYPETENSION | HPT | HTN | HIGH BLOOD PRESS* | BLOOD PRESSURE HIGH | HIGH DIASTOLIC BP | DIASTOLIC BP HIGH | ^ DIASTOLIC BP | HIGH DBP | HIGH SYSTOLIC BP | SYSTOLIC BP HIGH | ^ SYSTOLIC BP | HIGH SBP | HIGH SYS BP | BP HIGH | BP-HIGH | HIGHT BP | HBP | ^BP | HIGH BP | BP TOO HIGH | BP TOOOO HIGH | BP CHECK - HIGH | BP CHECK - STILL HIGH | BP CHECK, HIGH | CHECK BP, HIGH | BP REVIEW - STILL HIGH | BP STILL HIGH | BP STILL TOO HIGH | BP RETURNING UP HIGH

Coded as diagnosis of hypertension if the diagnosis, reason for encounter or reason for prescription fields stated the following:

HYPERT | HT

Excluded if the diagnosis, reason for encounter or reason for prescription fields contained the following terms indicating hypertension other than essential or primary hypertension:

OCULAR | OCCULAR | OPHTHALMIC | PORTAL | COAT | COLLAR | BORDER | HOUR | 24 HR | 24HR | HBPM | TIGHTNESS | PREGNANCY | GESTATIONAL | PARTUM | PULMONARY | PUMONARY | IDIOPATHIC | INTRACRANIAL | INTRA CRANIAL |INTRA-CRANIAL | INTRACANAL | INTRACRINIAL | CRANIAL | VENOUS HYPERT* | RENOVASCULAR

Excluded if the diagnosis, reason for encounter or reason for prescription fields contained the following terms indicating that hypertension referred to family history:

FAMIL* & HISTORY | FAM HIST | FH | FHX | PARENT | MOTHER | MUM| FATHER | DAD | PATERNAL | MATERNAL | HUSBAND | WIFE | DAUGHTER | BROTHER | SISTER

Excluded if the diagnosis, reason for encounter or reason for prescription fields contained the following terms indicating uncertainty around the diagnosis of hypertension:

?HTN | ?HPT | ? HYPERTENSION | ?HYPERTENSION |FEAR (OF) | PREVENTIVE CARE – HYPERTENSION | HYPERTENSION - PREVENTIVE CARE | RISK OF HYPERTENSION | RULE OUT HYPERTENSION | SUSPECTED HYPERTENSION | POSSIBLE HYPERTENSION | POSSIBLE HTN | POSSIBLY HYPERTENSION | NO HYPERTENSION | POSSIBLE EARLY HY | HYPERTENSION FOR INVESTIG | PRE HYPERTEN | PRE-HYPERTEN | LABILE

** Stata code is available from the first author upon request**
